# Health-related quality of life and associated factors in Chinese menstrual migraine patients: a cross-sectional study

**DOI:** 10.1186/s12905-022-01760-8

**Published:** 2022-05-15

**Authors:** Wenxiu Luo, Xing Cao, Jiayu Zhao, Jiaming Yang, Yu Cen, Jinlong He, Jing Luo, Yunling Zhong, Ying Luo, Xinyue Wang, Liqiu Yang, Xingyu Feng, Xiaoqing Pang, Jiazhu Zhang, Jiaming Luo

**Affiliations:** 1 Department of Neurology, The Affiliated Hospital of North Sichuan Medical College, Nanchong, Sichuan Province China; 2 Department of Psychiatry, The North Sichuan Medical College, Nanchong, Sichuan Province China; 3 Nanyang Centre for Public Administration, Nanyang Technological University, Singapore, Singapore

**Keywords:** Menstrual migraine, Health-related quality of life, Associated factors, SF-36

## Abstract

**Background:**

Menstrual migraine is a particular form of migraine with a significant impact on the quality of life for women afflicted. Presently, no study has reported the quality of life in menstrual migraine patients. This work aims to assess the health-related quality of life and identify its associated factors among Chinese menstrual migraine patients.

**Methods:**

The cross-sectional study group consisted of 109 patients with menstrual migraine, and the control group consisted of 397 female patients with non-menstrual migraine. In total, 506 patients completed questionnaires for demographic and clinical information, the Self-rating Idea of Suicide Scale, the Hamilton Depression Scale, the Hamilton Anxiety Scale, the Headache Impact Test-6, the Perceived Social Support Scale, the Pittsburgh Sleep Quality Index. Health-related quality of life was measured using the 36-Item Short Form Survey.

**Results:**

Compared with non-menstrual migraine patients, five dimensions of health-related quality of life were all found to be significantly impaired in menstrual migraine patients. Headache frequency (ß = − 0.218, *P* = 0.014), the impact of headache on daily life (ß = − 0.270, *P* = 0.002), depression symptoms (ß = − 0.345, *P* < 0.001) were significantly associated with physical component summary, depression symptoms (ß = − 0.379, *P* < 0.001), social support (ß = 0.270, *P* < 0.001), suicidal ideation (ß = − 0.344, *P* < 0.001) were closely related to mental component summary.

**Conclusion:**

Menstrual migraine patients had a significantly poorer health-related quality of life in many domains than non-menstrual migraine patients. Headache frequency, the impact of headache on daily life, depression symptoms, social support, and suicidal ideation were significantly associated with health-related quality of life in menstrual migraine patients.

*Trial registration*: ChiCTR1800014343. This study was registered prospectively on 7 January 2018 at Chinese Clinical Trial registry. http://www.chictr.org.cn/showproj.aspx?proj=24526

**Supplementary Information:**

The online version contains supplementary material available at 10.1186/s12905-022-01760-8.

## Background

Migraine is a common debilitating headache disorder that was ranked as the second-highest cause of disability in the 2016 Global Burden of Disease study [1]. Menstrual migraine (MM) is a subclass of migraine that can be classified into two types: pure menstrual migraine and menstrually-related migraine [2], and the prevalence of MM in female migraine patients is nearly 60% [3]. Compared with non-menstrual Migraine (NMM), MM is characterised by longer duration, greater frequency, less responsive to treatment, and may cause more severe disability and heavier life burden to patients [4, 5].

Health-related quality of life is defined as an individual's subjective perception of the impact of disease and treatment on physical, psychological as well as social and somatic domains of functioning and well-being [6]. HRQoL assessments could provide patients, researchers, and policymakers with information about the status of a patient’s health efficiently [7]. Limited number of studies indicate a negative effect of migraine on quality of life [8, 9]. In clinical practice, it has also been observed that a considerable proportion of MM patients suffer from anxiety and depression symptoms, sleep disorders, and other unpleasant mental and physical experiences, leading to decreased quality of life. Therefore, it is of great significance to actively search for and devote to improving controllable risk factors for MM patients. Clinicians have found that demographic factors such as age, clinical factors such as frequency and severity of pain, and psychological factors such as anxiety and depression symptoms play an important role in decreasing the migraine patients’ quality of life. However, studies on the quality of life and its associated factors in patients with MM are scarce. Based on the particularities of MM, further study of factors related to quality of life with MM is warranted.

The purpose of this cross-sectional study was to explore the HRQoL of MM versus NMM and to identify its associated factors among Chinese MM patients. It is hypothesized that (1) MM patients have lower HRQoL when compared with NMM; (2) Some clinical and psychological factors could affect the HRQoL of MM patients. Our research would provide a more comprehensive understanding of the HRQoL and associated factors among Chinese MM patients. Understanding these factors may help the development of individualized strategies for patients, thereby raising the HRQoL and reducing the financial, social and psychological burden.

## Method

### Participants and study design

The study was part of a cross-sectional study conducted from February 2019 to February 2020 at the neurology department of a hospital in western China. A total of 545 female migraine patients who met the diagnostic criteria were screened, and 39 persons who did not complete the HRQoL scale were excluded (3 with MM, 36 with NMM). A total of 506 female patients diagnosed with MM (n = 109) and NMM (n = 397) were included in this study, the inclusion criteria are in line with the International Classification of Headache Disorders, third Edition (ICHD-3) [2] developed by the Headache Classification Committee of the International Headache Society (Two experienced neurologists confirmed all diagnoses using ICHD-3 diagnostic criteria). Exclusion criteria: 1. Patients who had been diagnosed with psychiatric illness before the headache occurred, including anxiety disorder, depressive disorder, sleep disturbance, etc.; 2. Patients with secondary headaches; 3. patients suffered from other diseases that affect the quality of life; 4. patients who could not understand the questionnaire's content or did not complete the questionnaire; 5. Patients who refuse to sign informed consent. This survey was developed for this study, and the full questionnaire (English language version) is available as a supplementary file (Additional file 1). All participants signed an informed consent, and this study was approved by the Survey Ethics Committee of the first author’s affiliated institution. All authors had full access to all data.

## Data collection

### Demographic and headache information

Demographic variables include age, ethnicity (Han/minority), height (cm), weight (kg), body mass index (BMI), BMI is calculated as BMI = body mass (kg)/ (height (m))^2^. Headache related information includes headache frequency (the average number of days with headache per month over the last three months), duration (< 24 h/24–72 h/ > 72 h), severity (mild/moderate/severe), with or without family History and with or without aura.

### Health-related quality of life

HRQoL was measured using the Mandarin version of 36-Item Short Form Survey (SF-36) which has been tested with satisfactory reliability and validity [10, 11]. The SF-36 is a widely used survey of self-reported physical and mental health with 36 questions that measure 8 dimensions of health: physical functioning (PF), role-physical (RP), bodily pain (BP), general health (GH), vitality (VT), social functioning (SF), role-emotional (RE), and mental health (MH). The eight dimensions can also be summarizes as physical component summary (PCS) and mental component summary (MCS), which are average value of physical health dimensions (PF, RP, BP, GH) and mental health dimensions (VT, SF, RE, MH), respectively[7]. This scale was under license, and license was obtained for each survey.

### Impact of headache on daily life

We performed the Headache Impact Test-6 (HIT-6) to assess the impact of headaches on daily life. The HIT-6 is a concise and reliable tool for measuring headache burden based on six domains with a total score ranging from 36 to 78. The higher the score, the greater impact of headaches on daily activities[12]. This scale was under license, and license was obtained for each survey.

### Anxiety and depression symptoms

The severity of anxiety symptoms and depression symptoms of patients were assessed using the Hamilton Anxiety Scale (HAMA) and the Hamilton Depression Scale (HAMD) respectively. The higher the score, the more severe the anxiety or depression symptoms are [13, 14]. This scale was under license, and license was obtained for each survey.

### Social support

The Perceived Social Support Scale (PSSS) consists of 12 items, assessing perceived support from family, friends, and significant others. The total score ranges from 12 to 84, with higher scores representing higher perceived social support [15]. This scale was under license, and license was obtained for each survey.

### Sleep quality

We measured the sleep quality of patients using the Pittsburgh Sleep Quality Index (PSQI), a questionnaire that assesses subjectively perceived sleep quality over the last month. The PSQI is a self-report instrument that consists of 7 components: subjective sleep quality, sleep latency, sleep duration, habitual sleep efficiency, sleep disturbances, use of sleeping medications, and daytime dysfunction. The total score ranges from 0 to 21, with higher scores indicating poorer sleep quality [16]. This scale was under license, and license was obtained for each survey.

### Suicidal ideation

The self-rating Idea of Suicide Scale (SIOSS) is a self-report tool in Chinese with 26 questions that evaluate the suicide ideation of patients. We use the sum of three factors (despair, sleep, and optimism) to assess the level of suicidal ideation. The SIOSS has been shown to have good reliability and validity[17]. A higher score reflects a higher level of suicidal ideation. This scale was under license, and license was obtained for each survey.

### Data quality control

All the data collectors, including psychiatrists and neurologists, have received special data collection and management training to ensure uniformity in criteria evaluation.

### Statistical methods

Statistical analyses were performed with IBM SPSS version 26.0 software. Measurement data were expressed as Mean (standard deviation (SD)), and the t test was used to compare the two groups. Count data was presented as percentages (N, %), and the chi-square test was used to compare the two groups. Possible associated factors of PCS and MCS were analyzed using univariable logistic regression, stepwise multiple linear regression was performed on the factors with statistical significance in the univariate regression analyses to identify the independent factors that influenced PCS and MCS.* P* < 0.05 was considered statistically significant.

## Results

### Demographic and clinical characteristics

A total of 506 patients consented to participate in the study, including 109 MM patients and 397 NMM patients, with mean ages of 23.62 years (SD = 7.59) and 21.48 years (SD = 7.08), respectively. Table 1 presents the demographic and clinical characteristics of the participants and illustrates the differences between MM and NMM.

**Table 1:** Demographic and clinical data of our study sample

Variable	MM (N = 109)	NMM (N = 397)	*P* value
	Mean (SD)/ N (%)		
Age, years *	23.62 (7.59)	21.48 (7.09)	0.009
BMI	20.19 (2.50)	20.14 (2.38)	0.848
Headache frequency, days/month	3.97 (4.57)	3.83 (4.58)	0.750
PF*	89.13 (12.07)	91.52 (10.18)	0.037
RF*	59.17 (38.29)	69.40 (36.99)	0.012
BP	70.28 (14.28)	72.90 (14.95)	0.102
GH*	53.11 (17.21)	57.84 (17.74)	0.013
VT	61.24 (15.43)	62.01 (15.29)	0.641
SF*	79.68 (15.90)	93.78 (13.18)	< 0.001
RE	42.20 (39.19)	46.26 (40.77)	0.353
MH*	58.80 (15.08)	62.21 (14.80)	0.034
PCS*	67.82 (14.08)	72.91 (14.25)	0.001
MCS*	60.31 (16.45)	66.07 (15.54)	0.001
Ethnicity			0.131
Han	105 (96.33)	366 (92.19)	
Minority	4 (3.67)	31 (7.81)	
Family history			0.058
Yes	78 (71.56)	245 (61.71)	
No	31 (28.44)	152 (38.29)	
Aura			0.547
Yes	17 (15.60)	53 (13.35)	
No	92 (84.40)	344 (86.65)	
Degree of headache			0.141
Mild	32 (29.36)	148 (37.28)	
Moderate	60 (55.05)	209 (52.64)	
Severe	17 (15.59)	40 (10.08)	
Headache duration			0.539
< 24 h	99 (90.83)	372 (93.70)	
24-72 h	7 (6.42)	16 (4.03)	
> 72 h	3 (2.75)	9 (2.27)	

*Indicates statistically significant differences (*P* < 0.05) between the two groups

*BMI* body mass index, *PF* physical functioning, *RP* role-physical, *BP* bodily pain, *GH* general health, *VT* vitality, *SF* social functioning, *RE* role-emotional, *MH* mental health, *PCS* physical component summary, *MCS* mental component summary

### Health-related quality of life

**Fig. 1:**
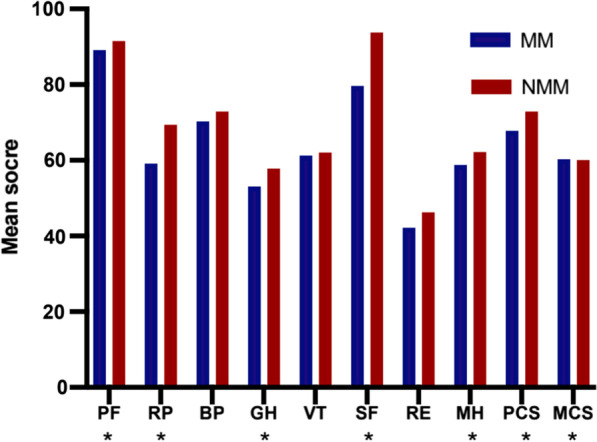
Mean scores in SF-36 for menstrual migraine (MM) patients vs. non-menstrual migraine (NMM), PF, physical functioning; RP, role-physical; BP, bodily pain; GH, general health; VT: vitality; SF: social functioning; RE: role-emotional; MH, mental health; PCS, physical component summary; MCS: mental component summary. *P < 0.05

Figure 1 presents the mean scores of different dimensions of the HRQoL among the MM and NMM groups. MM patients had significantly lower mean scores for the five dimensions of HRQoL (PF, RF, GH, SF, MH) compared with NMM patients. The average PCS and MCS scores of MM patients were 67.82(14.08) and 67.82(14.08), respectively, which were significantly lower than those of NMM patients. Detailed data are presented in Table 1.

### Univariate analysis of factors associated with PCS and MCS

Univariate analysis showed that the PCS and MCS scores were both correlated with headache frequency, anxiety and depression symptoms, suicidal ideation, sleep quality and social support, while the impact of headache on daily life was only related to PCS scores. Detailed data are presented in Table 2.

**Table 2:** Univariate linear of factors associated with SF-36

Variable	ß	t Value	95%CI	*P* Value
PCS				
Headache frequency	− 0.364	− 0.405	(− 1.671, − 0.572)	< 0.001
HIT-6	− 0.359	− 3.977	(− 0.719, − 0.241)	< 0.001
HAMD	− 0.399	− 4.507	(− 1.058, − 0.412)	< 0.001
HAMA	− 0.400	− 4.513	(− 1.073, − 0.418)	< 0.001
PSSS	0.260	2.786	(0.091, 0.543)	0.006
PSQI	− 0.335	− 3.658	(− 2.279, − 0.677)	< 0.001
SIOSS	− 0.360	− 3.989	(− 1.869, − 0.628)	< 0.001
MCS				
Headache frequency	− 0.244	− 2.607	(− 1.547, − 0.211)	0.010
HIT-6	− 0.151	− 1.585	(− 0.533, − 0.059)	0.116
HAMD	− 0.600	− 7.760	(− 1.619, − 0.960)	< 0.001
HAMA	− 0.459	− 5.351	(− 1.371, − 0.630)	< 0.001
PSSS	0.473	5.553	(0.433, 0.914)	< 0.001
PSQI	− 0.306	− 3.312	(2.517, − 0.632)	0.001
SIOSS	− 0.630	− 8.382	(− 3.155, − 1.948)	< 0.001

*PCS* physical component summary, *HIT-6* Headache Impact Text-6, *HAMD* Hamilton Rating Scale for Depression, *HAMA* Hamilton Rating Scale for Anxiety, *PSSS* Perceived social support scale, *PSQI* Pittsburgh Sleep Quality Index, *SIOSS* Self-rating Idea of Suicide Scale, *MCS*: mental component summary

### Step-wise multiple linear regression of factors associated with PCS and MCS

Headache frequency (ß = − 0.218, *P* = 0.014), depression symptoms (ß =− 0.345, *P* < 0.001), the impact of headache on daily life (ß = − 0.270, *P* = 0.002) were negatively associated with PCS, depression symptoms (ß = − 0.379, *P* < 0.001), suicidal ideation (ß = − 0.344, *P* < 0.001) were negatively associated with MCS, while social support (ß = 0.270, *P* < 0.001) was positively associated with MCS. Detailed data are presented in Table3.

**Table3:** Step-wise multiple linear regression of factors associated with SF-36

Variable	ß	t Value	95%CI	*P* Value
PCS				
Headache frequency	− 0.218	− 2.503	(− 1.198, − 0.139)	0.014
HIT− 6	− 0.270	− 3.151	(− 0.599, − 0.136)	0.002
HAMD	− 0.345	− 4.164	(− 0.936, − 0.332)	< 0.001
MCS				
HAMD	− 0.379	− 5.134	(− 1.124, − 0.498)	< 0.001
PSSS	0.270	3.912	(0.190, 0.581)	< 0.001
SIOSS	− 0.344	− 4.434	(− 2.018, − 0.771)	< 0.001

*PCS* physical component summary, *HIT-6* Headache Impact Text-6, *HAMD* Hamilton Rating Scale for Depression, *MCS* mental component summary, *PSSS* Perceived social support scale, *SIOSS* Self-rating Idea of Suicide Scale

## Disussion

This study provides, for the first time, a comprehensive assessment of the HRQoL and its associated factors in Chinese patients with MM. We found that the HRQoL in MM patients is lower in five dimensions of HRQoL (PF, RF, GH, SF, MH) than NMM patients. In addition, in the stepwise linear regression model, we found that PCS is related to headache frequency, depression symptoms, the impact of headache on daily life. MCS is related to depression symptoms, suicidal ideation, social support in MM patients. Such findings point the way to scientific strategies to improve HRQoL for MM patients.

Migraine increases the burden of patients [18], and seriously affects their life quality [8]. Nicodemo et al. used SF-36 to evaluate the life quality of MM, and found the scores of MM in the six dimensions of HRQoL (PF, RP, BP, GH, VT, SF)were lower than those of healthy women, and there is no significant difference in the scores of the two dimensions (RE, MH) [19]. Based on the severity and refractory of MM, we performed a subgroup analysis of migraine to compare the HRQoL in patients with MM and NMM. We found MM patients had lower mean scores for the five dimensions (PF, RF, GH, SF, MH) compared with NMM patients, and there were no statistically significant differences in scores for the three dimensions (BP, VT, RE). In addition, patients with MM scored lower than NMM for both PCS and MCS.

Our study demonstrated that depression symptoms are independently associated with PCS and MCS, after adjusting the confounding factors. Depression is a common comorbidity in migraine patients, and HRQoL was reduced in patients who had both migraine and depression relative to migraine patients who were not depressed [20]. A recent study in the United States found that depression symptoms are a predictor of headache frequency and migraine-related disability [21]. Hence, it is not difficult to understand that depression symptoms are closely associated with physical and mental health. In agreement with the study finding in Brazil, we demonstrated that the severity of depression symptoms is a predictor of HRQoL among MM [22]. Pradeep et al. reported the presence of depression was noted to add to the magnitude of migraine-related disability and diminished the quality of life in migraine patients, which is similar to the results of our study [23]. This study [23] also found that anxiety had a negative impact on the quality of life of patients who suffer from migraine. In our study, although univariate analysis showed that anxiety symptoms might be a significant risk factor for HRQoL in MM patients, no significant difference was detected by multivariate analysis. At present, it cannot be certain that anxiety symptoms are independently associated with HRQoL among MM patients.

MM patients who reported more frequent migraine attacks and greater impact of headaches on daily life could impair HRQoL by affecting their physical health. A downward trend in the quality of life of migraine was noted with the increased headache frequency [20]. Previous studies have proved that the more frequent migraine attacks and greater impact of headaches on daily life were predictors of detrimental effects on quality of life in migraine patients [23], which is similar to our results. In a recent study, Irimia found that a positive linear association between headache frequency and the risk of anxiety, depression in migraine patients [24]. Patients with monthly headache days ≥ 3 days are at higher risk of anxiety, while those with ≥ 19 days are at risk of depression. Moreover, patients with monthly headaches ≥ 10 days are often accompanied by severe disability [24]. It is interesting to note that patients experiencing only one to six headaches per year still show a reduction in quality of life, it might be due to the unpredictability of attacks that magnifies the effect of the few headache days on quality of life in a remarkable way [20]. Richard et al. [25] examined the association between headache-free days and the disease burden of migraine, and found headache-related disability shows a decreasing tendency with the headache-free days increasing [25].

Our research showed that the perception of social support is positively associated with MCS. An Italian study of chronic migraine patients with medication overuse found that social support is a predictor of the quality of life to some extent [26]. Moreover, a French study also found that the higher perceived social support was, the higher the probability of being an active consulter for migraine [27, 28]. Such headache counseling allowed patients to actively take prevention and treatment strategies to minimize the burden of migraine and relieve adverse emotions, and thus engaged more in social activities and improved HRQoL, especially in mental health-related HRQoL.

As the limitations of daily social and work-related activities caused by migraine, patients' mental health was impacted, and severe cases may lead to suicidal ideation. Our research found that suicidal ideation can affect MCS and could consequently predict HRQoL of MM patients. Many scholars agreed that migraine patients are associated with a poor quality of life and a higher likelihood of suicidal ideation [8, 9, 29, 30]. This study demonstrated, for the first time to our knowledge, the correlation between HRQoL and suicidal ideation among MM patients. Providing psychological treatment to MM patients with suicidal ideation may help to reduce suicide risk, as can the application of active treatment, improving their mental health and HRQoL.

As a clinical trigger for migraine, sleep disturbance can contribute to the vicious cycle [31]. Although sleep quality is associated with the PCS and MCS of patients with MM in the univariate analysis, it is no longer a significantly associated factor after adjusting for the potential confounders in the multivariate model. Thus, we cannot consider sleep quality as a predictor for HRQoL in MM patients. In the current study, the evidence describing the relationship between sleep quality and HRQoL was still insufficient among migraine patients [32], and this result was in line with the previous study.

## Conclusions

Considering HRQoL by migraine subtype, we found that MM patients had a worse HRQoL with statistically significantly lower mean scores in many domains than NMM patients. Depression symptoms are closely linked to physical and mental health in MM patients. For MM patients, PCS is related to the headache frequency and the impact of headaches on daily life, and MCS is related to social support and suicidal ideation. Moreover, sleep quality did not significantly impact the HRQoL among MM patients. The early application of individualized treatment may help improve the HRQoL in patients with MM.

There were some limitations to this study. The sample representativeness was limited because the sample was derived from the outpatient population of a hospital in southwest China. A multicenter study would be needed with a larger sample size and more associated factors to determine the factors that had an association with HRQoL of MM patients.

## Supplementary Information

**Additional file 1.** Headache basic situation questionnaire.

**Additional file 2.** Data and materials.

## Data Availability

The dataset supporting the conclusions of this article is included within the article and its additional file.

## Abbreviations

HRQoL: Health-related quality of life
MM: Menstrual migraine
NMM: Non-menstrual migraine
SIOSS: Self-rating idea of suicide scale
HAMD: Hamilton depression scale
HAMA: Hamilton anxiety scale
HIT-6: Headache impact test-6
PSSS: Perceived social support scale
PSQI: Pittsburgh sleep quality index
SF-36: 36-Item short form survey
ICHD-3: International classification of headache disorders, third edition
BMI: Body mass index
PF: Physical functioning
RP: Role-physical
BP: Bodily pain
GH: General health
VT: Vitality
SF: Social functioning
RE: Role-emotional
MH: Mental health
PCS: Physical component summary
MCS: Mental component summary
SD: Standard deviation
