# Supplementary material for: Health-related quality of life and associated factors in Chinese menstrual migraine patients: a cross-sectional study
Source: BMC Womens Health. 2022 May 15;22:177. doi: 10.1186/s12905-022-01760-8 (PMC9107648; doi:10.1186/s12905-022-01760-8)
Supplement: Supplementary file 1 — Additional file 1. Headache basic situation questionnaire. [file 12905_2022_1760_MOESM1_ESM.doc]

Headache basic situation

时间：

questionnaire

| **The general situation** | | | | | | | | | |
| --- | --- | --- | --- | --- | --- | --- | --- | --- | --- |
| Name |  | | | Gender | □man  □woman | Age |  | Nation |  |
| Height | cm | | | Weight | kg | BMI |  | | |
| Education | □Illiteracy □Primary school □Junior high school □High school or secondary school □College □Bachelor degree or above | | | | | | | | |
| **Past history** | | | | | | | | | |
| Past disease | |  | | | | | | | |
| **Headache situation** | | | | | | | | | |
| First headache age years；Headache course： （ years / months / days ） | | | | | | | | | |
| Headache and menstruation | | | □Irrelevant  □related -4++++-3 ++++ -2 ++++ -1 ++++ 1 ++++ 2 ++++ 3 ++++ 4 ++++ 5 | | | | | | |
| Duration of headache (unmedicated) | | | □﹤5s □5s-1min □1-10min □10-30min □30min-1h □1-2h □2-4h □4-12h  □12-24h □24-72h □more than 3 days （The time of general headache use√，The time of most serious headache use×） | | | | | | |
| The degree of headache | | | -0-----1-----2-----3-----4-----5-----6-----7-----8-----9-----10  No pain □Mild □Moderate □Severe | | | | | | |
| The average number of headaches per month in the last 3 months | | | □＜1day □1 □2 □3 □4 □5 □6 □7 □8 □9 □10 □11 □12 □13 □14 □15 □16 □17 □18 □19 □20 □21 □22 □23 □24 □25 □26 □27 □28 □29 □30 □Every day （Days of headache: √,Days of Migraine: **×**） | | | | | | |
| Migraine with aura | | | □No □Eyes flashing □Visual defect □Sensory disorder □aphasia □speech disorder □vertigo □tinnitus □Hearing loss □Diplopia □Confusion □Ataxia □hemiplegia  □Tired □inattention □Stiff neck □photophobia □Phonophobia □Blurred vision □Paled face | | | | | | |
| Family history of migraine | | | □No □Yes | | | | | | |

**HIT-6 Questionnaire**

INSTRUCTIONS : To complete, please circle one answer for each question.

1. When you have headaches, how often is the pain severe?

①Never——6分 ②Rarely——8分 ③Sometimes——10分 ④Very often——11 ⑤Always——13分

1. How often do headaches limit your ability to do usual daily activities including household work, work, school, or social activities?

①Never ②Rarely ③Sometimes ④Very often ⑤Always

1. When you have a headache, how often do you wish you could lie down?

①Never ②Rarely ③Sometimes ④Very often ⑤Always

4. In the past 4 weeks, how often have you felt too tired to do work or daily activities because of your headaches?

①Never ②Rarely ③Sometimes ④Very often ⑤Always

5. In the past 4 weeks, how often have you felt fed up or irritated because of your headaches?

①Never ②Rarely ③Sometimes ④Very often ⑤Always

1. In the past 4 weeks, how often did headaches limit your ability to concentrate on work or daily activities?

①Never ②Rarely ③Sometimes ④Very often ⑤Always

**Total Score：**
**SF-36 QUESTIONNAIRE**

**1.In general, would you say your health is:**

①Excellent-5 ②Very Good-4 ③Good-3 ④Fair-2 ⑤ Poor-1

1. **Compared to one year ago, how would you rate your health in general now**：

①Much better now than one year ago-5 ②Somewhat better now than one year ago -4 ③About the same-3 ④Somewhat worse now than one year ago -2⑤Much worse now than one year ago -1

**LIMITATIONS OF ACTIVITIES**

**3、The following items are about activities you might do during a typical day. Does your health now limit you in these activities? If so, how much?**

（1）Vigorous activities, such as running, lifting heavy objects, participating in strenuous sports.：

①Yes, Limited a lot-1 ②Yes, Limited a Little-2 ③No, Not Limited at all-3

（2）Moderate activities, such as moving a table, pushing a vacuum cleaner, bowling, or playing golf：

①Yes, Limited a lot-1 ②Yes, Limited a Little-2 ③No, Not Limited at all-3

1. Lifting or carrying groceries：

①Yes, Limited a lot-1 ②Yes, Limited a Little-2 ③No, Not Limited at all-3

1. Climbing several flights of stairs：

①Yes, Limited a lot-1 ②Yes, Limited a Little-2 ③No, Not Limited at all-3

1. Climbing one flight of stairs：

①Yes, Limited a lot-1 ②Yes, Limited a Little-2 ③No, Not Limited at all-3

1. Bending, kneeling, or stooping：

①Yes, Limited a lot-1 ②Yes, Limited a Little-2 ③No, Not Limited at all-3

1. Walking more than a mile：

①Yes, Limited a lot-1 ②Yes, Limited a Little-2 ③No, Not Limited at all-3

1. Walking several blocks：

①Yes, Limited a lot-1 ②Yes, Limited a Little-2 ③No, Not Limited at all-3

1. Walking one block：

①Yes, Limited a lot-1 ②Yes, Limited a Little-2 ③No, Not Limited at all-3

1. Bathing or dressing yourself：

①Yes, Limited a lot-1 ②Yes, Limited a Little-2 ③No, Not Limited at all-3

4、During the past 4 weeks, have you had any of the following problems with your work or other regular daily activities as a result of your physical health?

# （1）Cut down the amount of time you spent on work or other activities ①Yes-1 ②No-2

# （2）Accomplished less than you would like ①Yes ②No

# （3）Were limited in the kind of work or other activities ①Yes ②No

# （4）Had difficulty performing the work or other activities (for example, it took extra effort) ①Yes ②No

**5、**During the past 4 weeks, have you had any of the following problems with your work or other regular daily activities as a result of any emotional problems (such as feeling depressed or anxious)?

# Cut down the amount of time you spent on work or other activities ①Yes-1 ②No-2

# Accomplished less than you would like ①Yes ②No

# Didn't do work or other activities as carefully as usual ①Yes ②No

**6.Emotional problems interfered with your normal social activities with family, friends, neighbors, or groups?**

①Not at all-5 ②Slightly-4 ③Moderately-3 ④Severe-2 ⑤Very Severe-1

**7.How much bodily pain have you had during the past 4 weeks?**

①None-6 ②Very Mild-5.4 ③Mild-4.2 ④Moderate-3.1 ⑤Severe-2.2 ⑥Very Severe-1

# 8.During the past 4 weeks, how much did pain interfere with your normal work (including both work outside the home and housework)?

①Not at all-5 ②A little bit-4 ③Moderately-3 ④Quite a bit-2 ⑤Extremely-1

**9.These questions are about how you feel and how things have been with you during the last 4 weeks. For each question, please give the answer that comes closest to the way you have been feeling.**

# (1)Did you feel full of pep?

①All of the time-6 ②Most of the time-5 ③A good Bit of the Time-4 ④Some of the time-3

⑤A little bit of the time-2 ⑥None of the Time-1

# (2)Have you been a very nervous person?

①All of the time-1 ②Most of the time-2 ③A good Bit of the Time-3 ④Some of the time-4

⑤A little bit of the time -5 ⑥None of the Time-6

# (3)Have you felt so down in the dumps that nothing could cheer you up?

①All of the time-1 ②Most of the time-2 ③A good Bit of the Time-3 ④Some of the time-4

⑤A little bit of the time -5 ⑥None of the Time-6

# (4)Have you felt calm and peaceful?

①All of the time-6 ②Most of the time-5 ③A good Bit of the Time-4 ④Some of the time-3

⑤A little bit of the time-2 ⑥None of the Time-1

# (5)Did you have a lot of energy?

①All of the time-6 ②Most of the time-5 ③A good Bit of the Time-4 ④Some of the time-3

⑤A little bit of the time-2 ⑥None of the Time-1

# (6)Have you felt downhearted and blue?

①All of the time-1 ②Most of the time-2 ③A good Bit of the Time-3 ④Some of the time-4

⑤A little bit of the time -5 ⑥None of the Time-6

# (7)Did you feel worn out?

①All of the time-1 ②Most of the time-2 ③A good Bit of the Time-3 ④Some of the time-4

⑤A little bit of the time -5 ⑥None of the Time-6

# (8)Have you been a happy person?

①All of the time-6 ②Most of the time-5 ③A good Bit of the Time-4 ④Some of the time-3

⑤A little bit of the time-2 ⑥None of the Time-1

# (9)Did you feel tired?

①All of the time-1 ②Most of the time-2 ③A good Bit of the Time-3 ④Some of the time-4

⑤A little bit of the time -5 ⑥None of the Time-6

1. **During the past 4 weeks, how much of the time has your physical health or emotional problems interfered with your social activities (like visiting with friends, relatives, etc.)**

①All of the time-1 ②Most of the time-2 ③A good Bit of the Time-3 ④Some of the time-4

⑤A little bit of the time -5 ⑥None of the Time-6

**10.How true or false is each of the following statements for you?**

**(1)I seem to get sick a little easier than other people**

①Definitely true-1 ②Mostly true-2 ③Don't know-3 ④Mostly false-4 ⑤Definitely false-5

# (2)I am as healthy as anybody I know

①Definitely true-5 ②Mostly true-4 ③Don't know-3 ④Mostly false-2 ⑤Definitely false-1

# (3)I expect my health to get worse

①Definitely true-1 ②Mostly true-2 ③Don't know-3 ④Mostly false-4 ⑤Definitely false-5

# ()My health is excellent

①Definitely true-5 ②Mostly true-4 ③Don't know-3 ④Mostly false-2 ⑤Definitely false-1

# Pittsburgh Sleep Quality Index (PSQI)

Instructions:

The following questions relate to your usual sleep habits during the past month only. Your answers should indicate the most accurate reply for the majority of days and nights in the past month. Please answer all questions.


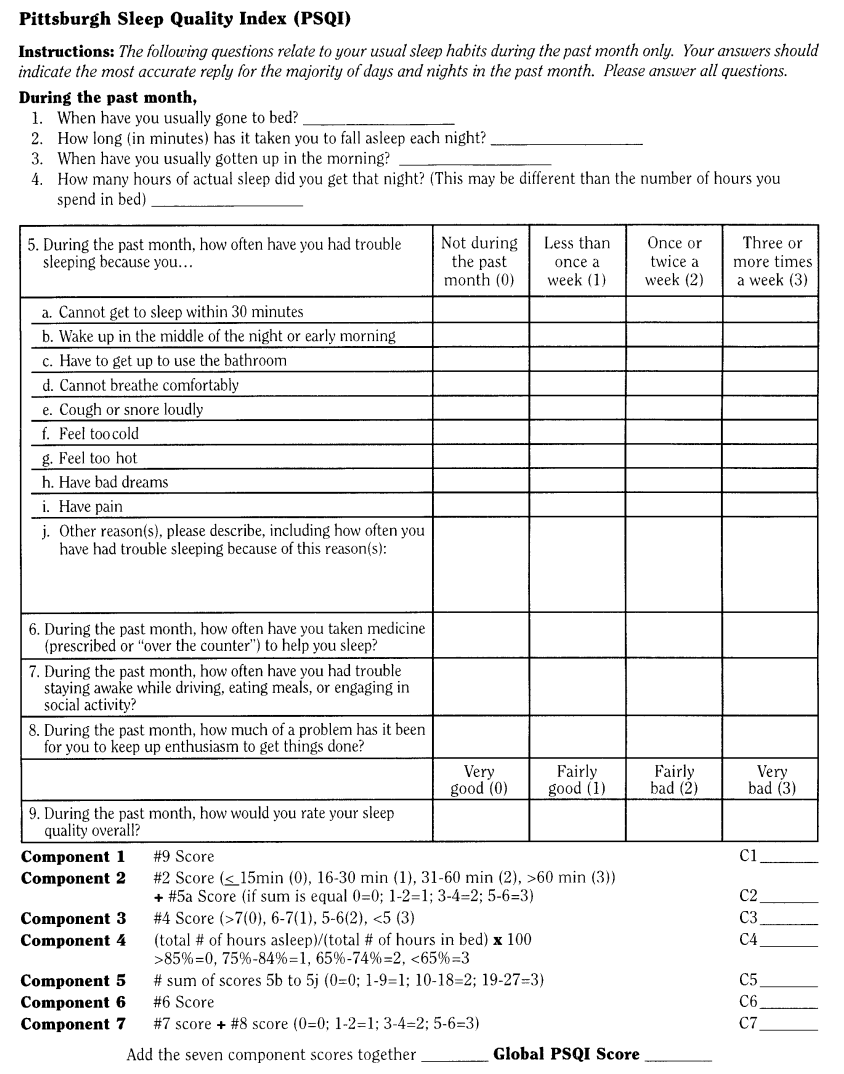


**HAMILTON DEPRESSION SCALE(HAMD)**

1. **Depressed mood (Sadness, hopeless, helpless, worthless)**

0-Absent

1-These feeling states indicated only on questioning

2-These feeling states spontaneously reported verbally

3-Communicated non-verbally - i.e. facial

expression, posture, voice, andtendency to weep

4-Patients reports virtually only; these feeling states in his spontaneous verbal and non-verbal communication

1. **Feelings of guilt:**

0-Absent

1-Self-reproach, feels he has let people down

2-Ideas of guilt or rumination over past errors or sinful deeds

3-Present illness is a punishment. Delusions of guilt

4-Hears accusatory or denunciatory voices and/or experiences threatening visual hallucinations

1. **Suicide:**

0-Absent

1- Feels life is not worth living

2-Wishes he were dead or any thoughts of possible death to self

3-Suicidal ideas or gesture

4-Attempts at suicide

1. **Insomnia early**

0-No difficulty falling asleep

1-Complains of occasional difficulty falling asleep i.e., more than1/2 hour

2-Complains of nightly difficulty falling asleep

1. **Insomnia middle**

0-No difficulty

1-Complains of being restless and disturbed during the night

2-Waking during the night - any getting out of bed (except to avoid)

1. **Insomnia late**

0-No difficulty

1-Waking in early hours of morning but goes back to sleep

2-Unable to fall asleep again if gets out of bed

1. **Work and activities**

0-No difficulty

1-Thoughts and feelings of incapacity, fatigue or weakness related to activities, work or hobbies

2-Loss of interest in activity, hobbies or work - by direct report of the patient or indirect in listlessness, indecision and vacillation (feels he has to push self to work or activities)

3-Decrease in actual time spent in activities or decrease in productivity. In hospital, spends less than 3 hours/day in activities (hospital job or hobbies)exclusive of ward chores

4-Stopped working because of present illness. In hospital, no activities except ward chores, or fails to perform ward chores unassisted

1. **Retardation (slowness of thought and speech; impaired ability to concentrate; decreased motor activity):**

0-Normal speech and thought

1-Slight retardation at interview

2-Obvious retardation at interview

3-Interview difficult

4-Complete stupor

1. **Agitation (Rating based on observation during interview)**

0-None

1-Fidgetiness

2-Playing with hands, hair, etc.

3-Moving about, can't sit still

4-Hand-wringing, nail biting, hair-pulling, biting of lips

1. **Anxiety psychic**

0-Absent

1-Subjective tension and irritability

2-Worrying about minor matters

3-Apprehensive attitude apparent in face or speech

4-Fears expressed without questioning

1. **Anxiety somatic: (physiologic concomitants of anxiety, such as dry mouth, gas, indigestion, diarrhea, cramps, belching, heart palpitations, headaches, hyperventilating, sighing having to urinate frequently sweating):**

0-Absent

1-Mild

2-Moderate

3-Severe

4-Incapacitating

1. **Somatic symptoms gastrointestinal**

0-None

1-Loss of appetite but eating without Encouragement

2-Difficulty eating without urging

1. **Somatic symptoms general**

0-None

1-Heaviness in limbs, back or head. Backaches, headache, muscle aches. Loss of energy and fatiguability.

2-Any clear-cut symptom

1. **Genital symptoms (such as loss of libido, menstrual disturbances) :**

0-None

1-Mild

2-Severe

1. **Hypochondriasis**

0-Not present

1-Self-absorption (bodily)

2-Preoccupation with health

3-Frequent complaints, requests for help, etc.

4-Hypochondriacal delusions

1. **Loss of weight (Rating by history)**

0-No weight loss

1-Probable weight loss associated with present illness

2-Definite (according to patient) weight loss

3-Not assessed

1. **Insight (Rating based on observation)**

0-Acknowledges being depressed and ill or not currently depressed

1-Acknowledges illness but attributes cause to bad food, climate, over-work, virus, need for rest, etc

2-Denies being ill at all

1. **Diurnal variation**

A. Note whether symptoms are worse in morning or evening. If no diurnal variation, mark note:

0-No variation or not currently depressed

1-Worse in A.M.

2-Worse in P.M.

B. When present, mark the severity of the variation:

0-None

1-Mild

2-Severe

1. **Depersonalization and derealization (such as feelings of unreality and nihilistic ideas):**

0-Absent

1-Mild

2-Moderate

3-Severe

4-Incapacitating

1. **Paranoid symptoms**

0-None

1-Suspicious

2-Ideas of reference

3-Delusions of reference and persecution

4-Hallucinations, persecutory

1. **Obsessional and compulsive symptoms**

0-Absent

1-Mild

2-Severe

1. **Decreased ability**

0-None

1-Subjective experience indicated only on questioning

2-The decreased ability spontaneously reported verbally

3-Difficulty completing routine or personal hygiene without encouragement, guidance and reassurance

4-Personal hygiene needs help from others

1. **Despair**

0-None

1-Sometimes doubt "whether the situation will get better", but accept after explanation

2-Persistent feeling of "hopeless", accepted after explanation

3-Feeling discouraged, pessimistic, and disappointed about the future, which cannot be resolved after explanation

4-Spontaneous repetition of "I'm not getting better" and so on

1. **Inferiority**

0-None

1-Inferiority indicated only on questioning

2-Inferiority spontaneously reported verbally

3-The patient's initiative to say "I am worthless" or "I am inferior to others" is only the degree of difference from the rate 2

4-Reaches the level of delusion

**HAMILTON ANXIETY SCALE(HAMA)**

0-Not present 1-Mild 2-Moderate 3-Severe 4-Very Severe

1. Anxiety mood: Worries, anticipation of the worst, fearful anticipation, irritability
2. Tension: Feelings of tension, fatigability, startle response, moved to tears easily, trembling, feelings of restlessness, inability to relax
3. Fears: Of dark, of strangers, of being left alone, of animals, of traffic, of crowds
4. Insomnia: Difficulty in falling asleep, broken sleep, unsatisfying sleep and fatigue on waking, dreams, nightmares, night terrors
5. Intellectual: Difficulty in concentration, poor memory
6. Depressed mood: Loss of interest, lack of pleasure in hobbies, depression, early waking.
7. Somatic (Muscular) : Pains and aches, twitchings, stiffness, myoclonic jerks, grinding of teeth
8. Somatic (Sensory) : Tinnitus, blurring of vision, hot and cold flushes, feelings of weakness
9. Cardiovascular symptoms: Tachycardia, palpitations, pain in chest, throbbing of vessels, fainting feelings, sighing, dyspnea
10. Respiratory symptoms: Pressure or constriction in chest, choking feelings, dyspnea
11. Gastrointestinal symptoms: Difficulty in swallowing, wind, abdominal pain, burning sensations, abdominal fullness, nausea, vomiting, borborygmi, looseness of bowels, loss of weight, constipation
12. Genitourinary symptoms: Frequency of micturition, urgency of micturition, amenorrhea, menorrhagia, development of frigidity, premature ejaculation, loss of libido, impotence
13. Autonomic symptoms: Dry mouth, flushing, pallor, tendency to sweet, glddiness, tension headache, raising of hair
14. Behavior at interview: Fidgeting, restlessness or pacing, trernor of hands, furrowed brow, strained face, sighing or rapid respiration, facial pallor, swallowing, etc.

**Self-rating Idea Of Suicid Scale (SIOSS)**

There are 26 questions on this questionnaire, please read them carefully, and tick one of the "Yes" or "No" brackets after each item according to the actual situation.

1. My daily life is full of things that interest me------Yes( ) No( )
2. I am convinced that life is cruel to me------Yes( ) No( )
3. I always feel pessimistic and disappointed------Yes( ) No( )
4. I cry easily or want to cry------Yes( ) No( )
5. I fell asleep easily and had a good sleep------Yes( ) No( )
6. I lie sometimes------Yes( ) No( )
7. How wonderful to live in these colorful world-----Yes( ) No( )
8. I really lack self-confidence-----Yes( ) No( )
9. I get angry sometimes-----Yes( ) No( )
10. I always feel that life is valuable-----Yes( ) No( )
11. I want to die at most of the time-----Yes( ) No( )
12. I sleep restlessly and get woken easily-----Yes( ) No( )
13. Sometimes I also gossip-----Yes( ) No( )
14. Sometimes I feel like I'm really useless-----Yes( ) No( )
15. Occasionally I laugh when I hear a dirty joke-----Yes( ) No( )
16. My future seems hopeless-----Yes( ) No( )
17. I want to end my life-----Yes( ) No( )
18. Wake up too early-----Yes( ) No( )
19. I always feel like my life is a failure-----Yes( ) No( )
20. I always take things seriously-----Yes( ) No( )
21. I always hold hope for the future-----Yes( ) No( )
22. I use to commit suicide-----Yes( ) No( )
23. I sometimes feel like I'm going to collapse-----Yes( ) No( )
24. Sometimes I lose sleep from worry-----Yes( ) No( )
25. I have damaged or lost something of someone else-----Yes( ) No( )
26. Sometimes I want to die, but I'm conflicted-----Yes( ) No( )

**Multidimensional Scale of Perceived Social Support**

The scale was composed of 12 items, and the 7-level scoring method was used for each item (1 = strongly disagree, 7 = strongly agree) . All item scores were summed to give a total score.

1. There is a special person who is around when I am in need.

2. There is a special person with whom I can share my joys and sorrows.

3. My family really tries to help me.

4. I get the emotional help and support I need from my family.

5. I have a special person who is a real source of comfort to me.

6. My friends really try to help me.

7. I can count on my friends when things go wrong.

8. I can talk about my problems with my family.

9. I have friends with whom I can share my joys and sorrows.

10. There is a special persons in my life who cares about my feelings.

11. My family is willing to help me make decisions.

12. I can talk about my problems with my friends.
